# The I-frame vs. S-frame: how neoliberalism has led behavioral sciences astray

**DOI:** 10.3389/fpsyg.2023.1247703

**Published:** 2023-09-07

**Authors:** Marike Andreas, Samira Barbara Jabakhanji

**Affiliations:** Medical Faculty Mannheim, Division of Public Health, Social and Preventive Medicine, Center for Preventive Medicine and Digital Health, Heidelberg University, Mannheim, Germany

**Keywords:** neoliberalism, health policies, commentary, complex system (CS), interventions

## Abstract

In their recently published paper, Chater and Loewenstein critically elaborate on the differences between interventions that focus on individual behavior (‘i-frame’), as opposed to the systems in which health behavior occurs (‘s-frame’). They point out that behavioral scientists frequently rely on individual-level interventions, rather than systemic change to improve population health. As individual-level interventions have fallen short of the author’s expectations to fix health problems, the authors argue that behavioral scientists should focus more on system-level change. They warn behavioral scientists that by framing disease as an individual problem they hinder real change. We agree with the arguments made by the authors; nevertheless, we propose that bringing underlying causes for the i-frame focus to light would advance their argument. In our commentary, we discuss that neoliberalism might be a reason for the focus on individual interventions in behavioral health sciences.

## Introduction

To begin with, we would like to congratulate the authors Chater and Loewenstein for the publication of their recent paper ‘*The I-frame vs. s- frame: how focusing on individual-level solutions has led behavioral public policy astray*,’ which we regard as a highly valuable contribution to the fields of health research and healthcare governance. The relevance and richness of this paper inspired us to further delve into this subject and comment on the aforementioned paper.

In their paper, Chater and Loewenstein (2022) critically elaborate on the differences between interventions that focus on individual behavior (‘i-frame’), as opposed to the systems in which health behavior occurs (‘s-frame’). They point out that behavioral scientists frequently rely on individual-level interventions, rather than systemic change to improve population health. In the face of mounting evidence that individual-level interventions are less effective than structural interventions see for example the recent discussion on nudging (Maier et al. 2022), the authors argue that behavioral scientists should apply their skills to generating system-level change. Moreover, the authors warn behavioral scientists that by framing disease as an individual problem, they unwillingly support corporations and hinder real change. We agree with the arguments made by the authors; nevertheless, we propose that bringing underlying causes for the i-frame focus to light would advance their argument and help facilitate the development of more effective interventions.

As the authors state, most intervention research in behavioral science and health psychology has focused on individuals. This has also been observed in other health disciplines, including public health (Schrecker, 2016). For example, Popay et al. (2010) have called this phenomenon a “lifestyle drift,” the tendency to acknowledge systemic factors that influence health but to drift toward individual factors in health policy-making. Furthermore, Schrecker and Bambra (2015) reject the “sticking plaster” approach of trying to cure societal problems with individual interventions, such as diet counseling for people with obesity. Since researchers from various disciplines in the health sciences have observed this overemphasis on the role of individual behavior, we propose that there might be a common explanation for this phenomenon.

### Understanding reasons for the i-frame focus

On the surface, the focus on the i-frame might be due to the practicability of i-frame approaches and the hierarchy of evidence in healthcare that favors interventions that can be tested with randomized-controlled trials, as the authors argue (p.5). In contrast, changes on the systemic level need to be assessed with natural experiment designs, which are not seen as the gold standard for evidence. Another explanation by the authors is that corporations promote i-framing to prevent the negative consequences of structural change for industry actors, such as fiscal measures or advertising bans (p.9). For example, the nutrition industry tends to frame obesity as a personal responsibility (Jenkin et al., 2011), which leads to an emphasis on educational obesity interventions. We perceive that in their argument, the authors reproduce the same phenomenon they are criticizing: seeking i-frame explanations for a phenomenon that is caused on a systemic level. We suggest that the focus on individual-level interventions is not only the creation of companies profiting of this framing or researchers seeking to identify practical and evidence-based solutions to real-world problems, but that it reflects the structural organization of society today.

A trend toward individual health promotion has been observed since the 1980s (Baggott, 1991; Burrows et al., 1995) and has coincided with the rise of neoliberal political ideology in the Global North. We propose that this is not accidental, but that the individualization of health research might be the result of neoliberalism. Here, we use the term neoliberalism to describe an ideology, rather than an economic concept. At the heart of neoliberal ideology is the principle of minimal government intervention (Ayo, 2012); the idea that markets, not the government, will solve societal problems by reacting to a demand through the creation of new markets and consumable goods. Following this logic, responsible individuals should consume these new goods in order to maintain health (Ayo, 2012). According to neoliberal thinkers, individuals are “free to choose” in the marketplace (Friedman, 1990). Health risks are thus individualized, and unhealthy behavior becomes a personal responsibility detached from the structural conditions shaping it (Spindler, 2010; Gollust and Lynch, 2011; ILO, 2011). As a consequence, lifestyles and related health outcomes can become subject to blame-shifting, stigmatization and discrimination (ILO, 2011) or reward systems (such as through insurance companies), while the role of governments to create equal opportunities to health is being neglected. In the words of Hayek (1960, p.71), one of the founding fathers of neoliberalism, “Liberty not only means that the individual has both the opportunity and the burden of choice; it also means that [they] must bear the consequences of [their] actions and will receive praise or blame for them.” Following this neoliberal logic, the less effective behavioral i-frame interventions become the instrument of choice as they aim to change individual behavior without directly affecting the system within which individuals operate.

### Ideology shapes research

Understanding the political ideology shaping health research today is an important first step toward designing impactful interventions on the systemic level. As Baggott (1991) argues “public health reforms will only succeed if the reformers themselves operate with full awareness of the political dimension” (p. 191). It seems that behavioral scientists, especially in the health sciences, pay little attention to the political context in which health behaviors are shaped and researched. However, only if we understand the circumstances that shape health behavior can we create successful interventions to promote better and more equitable health outcomes. Thus, while we fully support the argument of Chater and Loewenstein to focus more on s-frame interventions, we propose that it would benefit from an analysis of the ideology that favors individual interventions, namely neoliberalism (even though we would be open to other explanations by the authors). The “influential line of thinking” (p. 2) that the authors themselves admit to have subscribed to in the past might simply be neoliberal ideology.

Although often unacknowledged, neoliberalism is the dominant political ideology (aka, “line of thinking,” p. 2) of our time (Monbiot, 2016). It is the system in which health researchers work and which creates health and wellbeing as well as disease and death. As described, the focus on individual behavior is inherent in neoliberalism, which leads to an oversimplification of complex health phenomena. Most recently, debates on mandatory mask-wearing to protect against COVID-19 infection have not yielded binding legislation, for example in Germany, supporting the idea of neoliberal influences in health policy. However, our impression is that neoliberalism has had little attention in the psychological and behavioral sciences over the past decade. While some authors have examined the influence of neoliberal ideology on health policy (Navarro, 2008; Ayo, 2012; Schrecker and Bambra, 2015; Lopez et al., 2022), to our knowledge, none have investigated how it impacts behavioral research.

Investigating neoliberal impacts on behavioral research appears like a fruitful area for future study and a pathway to creating effective interventions for population health. Prior studies have found that ideologies influence how we frame health problems (Russell et al., 2020) and the solutions that seem available to fix them (Navarro, 2008). By acknowledging neoliberal impacts on behavioral research, the limitations of how neoliberalism views health behavior may also become more evident. With this understanding, behavioral researchers may be encouraged to move away from the narrative of individual responsibility toward developing interventions that consider systemic factors. Simultaneously, this could contribute to the development of scientifically robust interventions that are better tailored to the cultural and social contexts of the populations whose health they aim to improve. Consequently, we agree with Chater and Loewenstein’s argument to sufficiently acknowledge contextual factors when researching in an i-frame paradigm. With a crucial contextual factor being the political and economic environment, we encourage researchers to reflect upon the ideological influences shaping their research.

### Shifting behavioral research to s-frame thinking

To add to Chater and Loewenstein‘s elaborations, we suggest that behavioral scientists extend the frameworks they have been using this far, to incorporate variables from the social, political, environmental and economic dimensions. When we look at the way traditional theories of behavior change have been constructed, socio-demographic, economic and environmental variables are missing (Whitmee et al., 2015). Incorporating systemic factors of behavior change in behavioral science theories and interventions may encourage s-frame thinking. More recently, approaches of complex systems thinking investigate behaviors as emergent outcomes of dynamic interactions within intricate systems (Keshavarz Mohammadi, 2019). For example, the Complex Systems Framework for Obesity (Griffiths et al., 2023) acknowledges the need to consider system boundaries, inter-relationships of different parts of the system, and different stakeholder perspectives in obesity interventions. Recommended tools include “system dynamics modelling (social-) network analysis, and agent-based modelling (Griffiths et al., 2023).” These approaches can help behavioral scientists to understand how various elements within the social, political, economic, and environmental realms coalesce to shape behaviors. Already at the planning stage, scientists should consider the evaluation of health interventions on social, environmental, economic, and ideological dimensions to achieve lasting and meaningful change (Vergunst et al., 2019). This might be aided by working with and learning from disciplines with more critical approaches to health behavior, such as public health or sociology.

## Discussion

Chater and Loewenstein highlighted the important overemphasis on i-frame interventions and the missing focus on s-frame interventions that take contextual factors more into account. We propose that traditional theories of behavior change should no longer neglect systemic variables. The authors mentioned interdisciplinarity as a possible mechanism for bringing the s-frame into focus, which reflects the limited role interdisciplinarity has played in behavioral science to date. We agree that a broader perspective can be attained by collaborating with various disciplines from the social and health sciences, if leading to an integration of complex systems thinking into the behavioral science discipline itself. Moreover, we have highlighted the more critical perspective on individualistic thinking specifically of public health researchers above. We emphasize that behavioral scientists can learn from the ongoing discussion on upstream versus downstream interventions in the field of public health. Acknowledging the rich debate on s- versus i-frame interventions that has been led in public health by scientists such as Vincente Navarro, Clare Bambra and Ted Schrecker would strengthen the argument made by Chater and Loewenstein.

In summary, we argue that promoting s-frame-thinking in behavioral science means critically questioning the i-frame focus in a neoliberal capitalist system, learning from other disciplines and considering systemic variables in traditional theories of behavior change.

## Data availability statement

The original contributions presented in the study are included in the article/supplementary material, further inquiries can be directed to the corresponding author.

## Author contributions

All authors listed have made a substantial, direct, and intellectual contribution to the work and approved it for publication.

## Conflict of interest

The authors declare that the research was conducted in the absence of any commercial or financial relationships that could be construed as a potential conflict of interest.

## Publisher’s note

All claims expressed in this article are solely those of the authors and do not necessarily represent those of their affiliated organizations, or those of the publisher, the editors and the reviewers. Any product that may be evaluated in this article, or claim that may be made by its manufacturer, is not guaranteed or endorsed by the publisher.
